# Supplementary material for: Adopting and validating a technology acceptance model-based paradigm to assess acceptance and satisfaction with electronic health information system by healthcare providers in resource-limited governmental and non-governmental hospitals
Source: PLOS Digit Health. 2026 Apr 6;5(4):e0001343. doi: 10.1371/journal.pdig.0001343 (PMC13052840; doi:10.1371/journal.pdig.0001343)
Supplement: S2 Table — (DOCX) [file pdig.0001343.s002.docx]

**S2 Table.** Operationalization of constructs and indicators for EHIS acceptance and satisfaction – developed and adapted items

| **Construct** | **#** | **Item** |
| --- | --- | --- |
| Relative advantage  (RA) | 1 | Adopting EHIS improves the quality of my work. |
|  | 2 | By adopting EHIS, my work productivity has increased. |
|  | 3 | Adopting EHIS enhances my job effectiveness. |
|  | 4 | Using EHIS increases my job performance. |
|  | 5 | Using EHIS enables me to quickly and easily obtain information on investigation or treatment procedures. |
|  | 6 | Using EHIS improves patient safety. |
|  | 7 | Using EHIS reduces time and costs. |
| Compatibility  (COMP) | 1 | The current IT infrastructure supports the use of electronic EHIS. |
|  | 2 | Using EHIS fits well with my work style. |
|  | 3 | Using EHIS is fully compatible with my current situation. |
| Complexity  (CPLX) | 1 | I believe that EHIS is complicated to use. |
|  | 2 | It is difficult for me to remember how to perform tasks using EHIS. |
|  | 3 | Adopting EHIS requires a significant amount of mental effort. |
|  | 4 | Using EHIS is often frustrating. |
| Top management support  (TMS) | 1 | Top management support is important for adopting EHIS. |
|  | 2 | Support from related departments is important for adopting EHIS. |
|  | 3 | Management provided helpful support during the implementation of EHIS. |
|  | 4 | Management expects me to use EHIS. |
| IT support and training  (ITS) | 1 | I have received sufficient formal training to use EHIS. |
|  | 2 | The IT staff provided adequate support for EHIS. |
|  | 3 | I will use EHIS if I receive proper training. |
|  | 4 | I will use EHIS if I can obtain technical support. |
|  | 5 | There are enough workstations available for staff to use. |
|  | 6 | The training provided has given us confidence in using EHIS. |
| System quality  (SQ) | 1 | The information provided by EHIS is always accurate. |
|  | 2 | The information provided by EHIS is always timely. |
|  | 3 | I find the EHIS interface to be user-friendly. |
|  | 4 | The EHIS is stable and rarely experiences problems or crashes. |
| Perceived usefulness  (PU) | 1 | The electronic EHIS is integrated into my daily work. |
|  | 2 | The information provided by EHIS makes my work easier. |
|  | 3 | I have access to the information where I need it. |
|  | 4 | I have access to the information when I need it. |
|  | 5 | The information provided by EHIS is always updated. |
|  | 6 | The data I record are important for patient care. |
|  | 7 | I am confident in the reliability of the documented data. |
|  | 8 | Using EHIS avoids duplication of examinations. |
|  | 9 | Using EHIS reduces the risk of errors. |
| Perceived ease of use  (PEOU) | 1 | It is easy to learn how to use EHIS. |
|  | 2 | It is easy to use EHIS. |
|  | 3 | It is easy to understand how to perform the intended tasks using EHIS. |
|  | 4 | Learning to use EHIS will require a significant amount of time. |
| Behavioral intension  (BI) | 1 | When available in my clinical practice, I intend to use EHIS for all my clinical activities. |
|  | 2 | When available in my community, I intend to adopt EHIS for all my clinical activities. |
|  | 3 | The likelihood that I will use EHIS for all my clinical activities, when available in my organization, is very high. |
| Competitive pressure  (CP) | 1 | We are aware of EHIS implementation in our hospital. |
|  | 2 | We understand the competitive advantages offered by EHIS in our hospital. |

BI: behavioral intention, COMP: compatibility, CP: competitive pressure, CPLX: complexity, EHIS: electronic health information system, ITS: IT support and training, PEOU: perceived ease of use, PU: perceived usefulness, RA: relative advantage, SQ: system quality, TMS: top management support.
