# Supplementary material for: Adopting and validating a technology acceptance model-based paradigm to assess acceptance and satisfaction with electronic health information system by healthcare providers in resource-limited governmental and non-governmental hospitals
Source: PLOS Digit Health. 2026 Apr 6;5(4):e0001343. doi: 10.1371/journal.pdig.0001343 (PMC13052840; doi:10.1371/journal.pdig.0001343)
Supplement: S3 Table — (DOCX) [file pdig.0001343.s003.docx]

**S3 Table.** The study questionnaire

|  | **Demographic and practice variables** | |  |  |  |  |
| --- | --- | --- | --- | --- | --- | --- |
|  | Sex: | o  Male |  |  |  |  |
|  |  | o  Female |  |  |  |  |
|  | Educational qualification | o  Diploma |  |  |  |  |
|  |  | o  Undergraduate degree |  |  |  |  |
|  |  | o  Postgraduate degree |  |  |  |  |
|  | Age (years): |  |  |  |  |  |
|  | Years of practical experience in a hospital setting: |  |  |  |  |  |
|  | Duration of EHIS/computer usage (years): |  |  |  |  |  |
|  | Formal EHIS/computer training: | o  No |  |  |  |  |
|  |  | o  Yes |  |  |  |  |
|  | Job title: | o  Physicians |  |  |  |  |
|  |  | o  Nurses |  |  |  |  |
|  |  | o  Pharmacist |  |  |  |  |
|  |  | o  Laboratory technicians |  |  |  |  |
|  |  | o  Radiology technician |  |  |  |  |
|  |  | o  Medical Secretary |  |  |  |  |
|  | **Indicate the level of your agreement/disagreement on the following items:** | **Strongly disagree** | **Disagree** | **Neutral** | **Agree** | **Strongly agree** |
| 1 | Adopting EHIS improves the quality of my work. |  |  |  |  |  |
| 2 | By adopting EHIS, my work productivity has increased. |  |  |  |  |  |
| 3 | Adopting EHIS enhances my job effectiveness. |  |  |  |  |  |
| 4 | Using EHIS increases my job performance. |  |  |  |  |  |
| 5 | Using EHIS enables me to quickly and easily obtain information on investigation or treatment procedures. |  |  |  |  |  |
| 6 | Using EHIS improves patient safety. |  |  |  |  |  |
| 7 | Using EHIS reduces time and costs. |  |  |  |  |  |
| 8 | The current IT infrastructure supports the use of electronic EHIS. |  |  |  |  |  |
| 9 | Using EHIS fits well with my work style. |  |  |  |  |  |
| 10 | Using EHIS is fully compatible with my current situation. |  |  |  |  |  |
| 11 | I believe that EHIS is complicated to use. |  |  |  |  |  |
| 12 | It is difficult for me to remember how to perform tasks using EHIS. |  |  |  |  |  |
| 13 | Adopting EHIS requires a significant amount of mental effort. |  |  |  |  |  |
| 14 | Using EHIS is often frustrating. |  |  |  |  |  |
| 15 | Top management support is important for adopting EHIS. |  |  |  |  |  |
| 16 | Support from related departments is important for adopting EHIS. |  |  |  |  |  |
| 17 | Management provided helpful support during the implementation of EHIS. |  |  |  |  |  |
| 18 | Management expects me to use EHIS. |  |  |  |  |  |
| 19 | I have received sufficient formal training to use EHIS. |  |  |  |  |  |
| 20 | The IT staff provided adequate support for EHIS. |  |  |  |  |  |
| 21 | I will use EHIS if I receive proper training. |  |  |  |  |  |
| 22 | I will use EHIS if I can obtain technical support. |  |  |  |  |  |
| 23 | There are enough workstations available for staff to use. |  |  |  |  |  |
| 24 | The training provided has given us confidence in using EHIS. |  |  |  |  |  |
| 25 | The information provided by EHIS is always accurate. |  |  |  |  |  |
| 26 | The information provided by EHIS is always timely. |  |  |  |  |  |
| 27 | I find the EHIS interface to be user-friendly. |  |  |  |  |  |
| 28 | The EHIS is stable and rarely experiences problems or crashes. |  |  |  |  |  |
| 29 | The electronic EHIS is integrated into my daily work. |  |  |  |  |  |
| 30 | The information provided by EHIS makes my work easier. |  |  |  |  |  |
| 31 | I have access to the information where I need it. |  |  |  |  |  |
| 32 | I have access to the information when I need it. |  |  |  |  |  |
| 33 | The information provided by EHIS is always updated. |  |  |  |  |  |
| 34 | The data I record are important for patient care. |  |  |  |  |  |
| 35 | I am confident in the reliability of the documented data. |  |  |  |  |  |
| 36 | Using EHIS avoids duplication of examinations. |  |  |  |  |  |
| 37 | Using EHIS reduces the risk of errors. |  |  |  |  |  |
| 38 | It is easy to learn how to use EHIS. |  |  |  |  |  |
| 39 | It is easy to use EHIS. |  |  |  |  |  |
| 40 | It is easy to understand how to perform the intended tasks using EHIS. |  |  |  |  |  |
| 41 | Learning to use EHIS will require a significant amount of time. |  |  |  |  |  |
| 42 | When available in my clinical practice, I intend to use EHIS for all my clinical activities. |  |  |  |  |  |
| 43 | When available in my community, I intend to adopt EHIS for all my clinical activities. |  |  |  |  |  |
| 44 | The likelihood that I will use EHIS for all my clinical activities, when available in my organization, is very high. |  |  |  |  |  |
| 45 | We are aware of EHIS implementation in our hospital. |  |  |  |  |  |
| 46 | We understand the competitive advantages offered by EHIS in our hospital. |  |  |  |  |  |

EHIS: electronic health information system
