# Supplementary figures and images for: Adopting and validating a technology acceptance model-based paradigm to assess acceptance and satisfaction with electronic health information system by healthcare providers in resource-limited governmental and non-governmental hospitals
Source: PLOS Digit Health. 2026 Apr 6;5(4):e0001343. doi: 10.1371/journal.pdig.0001343 (PMC13052840; doi:10.1371/journal.pdig.0001343)

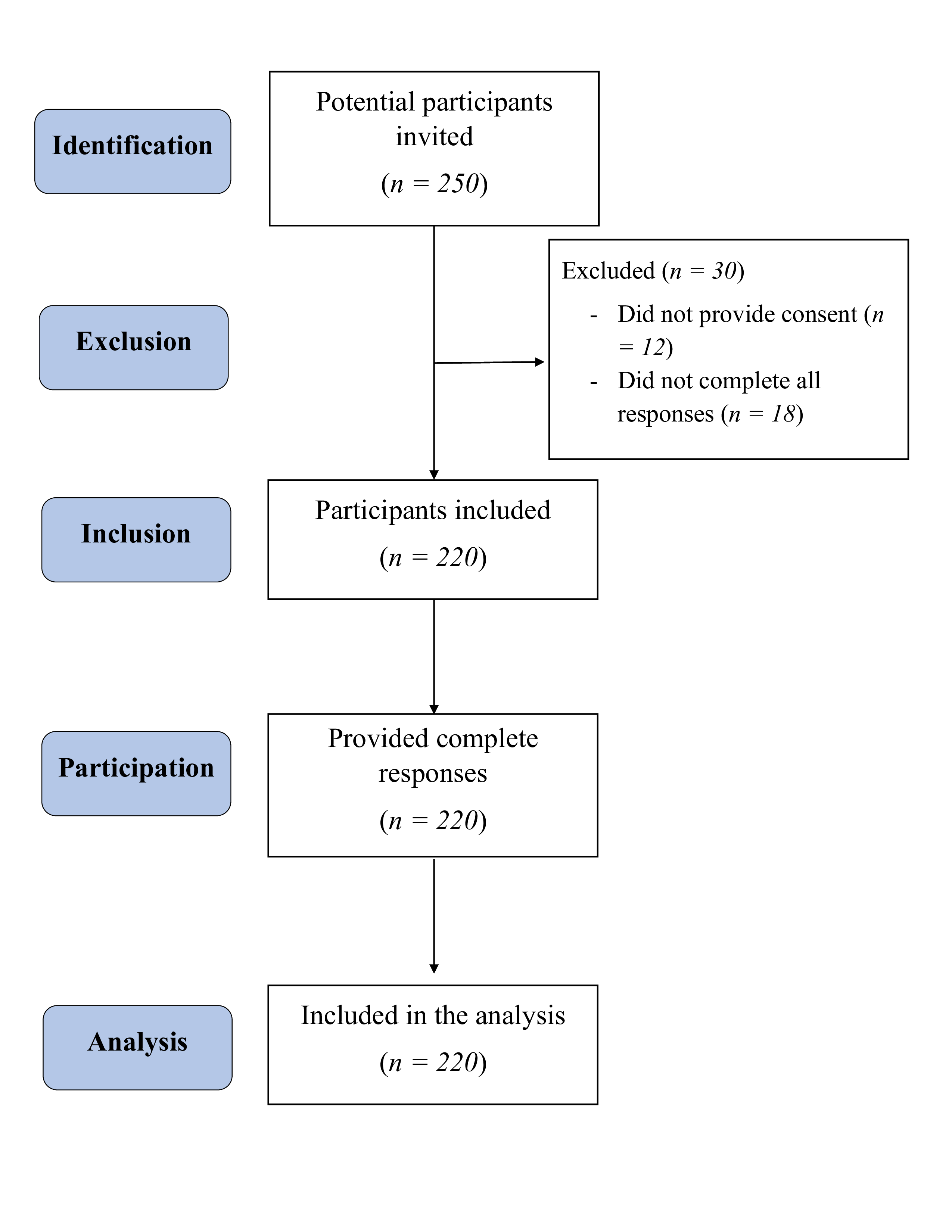

Supplement: S1 Fig — (TIFF) [file pdig.0001343.s004.tiff]
