# Supplementary material for: Adopting and validating a technology acceptance model-based paradigm to assess acceptance and satisfaction with electronic health information system by healthcare providers in resource-limited governmental and non-governmental hospitals
Source: PLOS Digit Health. 2026 Apr 6;5(4):e0001343. doi: 10.1371/journal.pdig.0001343 (PMC13052840; doi:10.1371/journal.pdig.0001343)
Supplement: S4 Table — (DOCX) [file pdig.0001343.s005.docx]

**S4 Table.** Variance inflation factor values

| **Item** | **Variance inflation factor** |
| --- | --- |
| RA1 | 2.7 |
| RA4 | 2.6 |
| RA5 | 2.0 |
| RA6 | 1.7 |
| RA7 | 2.0 |
| BI1 | 2.3 |
| BI2 | 2.3 |
| BI3 | 1.9 |
| CPLX1 | 3.0 |
| CPLX2 | 3.1 |
| CPLX3 | 2.5 |
| CP1 | 3.1 |
| CP2 | 3.1 |
| COMP1 | 1.8 |
| COMP2 | 3.1 |
| COMP3 | 2.6 |
| ITS1 | 1.5 |
| ITS2 | 1.6 |
| ITS3 | 1.4 |
| PEOU1 | 1.6 |
| PEOU2 | 2.0 |
| PEOU3 | 2.2 |
| PU1 | 2.5 |
| PU5 | 2.5 |
| PU6 | 2.3 |
| PU7 | 2.0 |
| PU8 | 2.3 |
| PU9 | 3.0 |
| SQ1 | 1.5 |
| SQ2 | 1.4 |
| SQ3 | 1.4 |
| TMS1 | 2.7 |
| TMS2 | 3.3 |
| TMS3 | 2.1 |
| TMS4 | 1.7 |

BI: behavioral intention, COMP: compatibility, CP: competitive pressure, CPLX: complexity, EHIS: electronic health information system, ITS: IT support and training, PEOU: perceived ease of use, PU: perceived usefulness, RA: relative advantage, SQ: system quality, TMS: top management support.
