# Supplementary material for: Adopting and validating a technology acceptance model-based paradigm to assess acceptance and satisfaction with electronic health information system by healthcare providers in resource-limited governmental and non-governmental hospitals
Source: PLOS Digit Health. 2026 Apr 6;5(4):e0001343. doi: 10.1371/journal.pdig.0001343 (PMC13052840; doi:10.1371/journal.pdig.0001343)
Supplement: S5 Table — (DOCX) [file pdig.0001343.s006.docx]

**S5 Table.** Standardized root mean square residual

|  | **Saturated model** | **Estimated model** |
| --- | --- | --- |
| **SRMR** | 0.075 | 0.079 |

SRMR: standardized root mean square residual
