# Supplementary material for: Adopting and validating a technology acceptance model-based paradigm to assess acceptance and satisfaction with electronic health information system by healthcare providers in resource-limited governmental and non-governmental hospitals
Source: PLOS Digit Health. 2026 Apr 6;5(4):e0001343. doi: 10.1371/journal.pdig.0001343 (PMC13052840; doi:10.1371/journal.pdig.0001343)
Supplement: Attachment — Submitted filename: Response to comments PLOS Digital Health.docx [file pdig.0001343.s008.docx]

**Submission ID:** PDIG-D-25-00865

**Title:** Adopting and validating a technology acceptance model-based paradigm to assess acceptance and satisfaction with electronic health information system by healthcare providers in resource-limited governmental and nongovernmental hospitals

**Journal:** PLOS Digital Health

**Dear Prof. Dr. Phat Huynh, Dear Prof. Dr. Leo Anthony Celi**

**Editor-in-Chief**

**PLOS Digital Health**

Thank you for sharing your comments and the comments of the reviewers on our submitted manuscript. We are very delighted to learn that you and the reviewers have found the manuscript timely, important, relevant, and interesting. We are also very happy to learn that you will accept the manuscript after addressing the comments of the reviewers and revise the manuscript accordingly.

Please note that we have found all comments and suggestions raised by the reviewers were legitimate and consideration worthy. In this revision, all comments and suggestions were considered and the manuscript was amended accordingly and as the reviewers suggested.

Please find below a detailed point-by-point account of how the comments were addressed and how the manuscript was revised accordingly. For your convenience and to expedite the decision, all changes were highlighted in red font.

**Editor’s comments**

1. **Comment:** Thank you for submitting your manuscript to PLOS Digital Health. After careful consideration, we feel that it has merit but does not fully meet PLOS Digital Health's publication criteria as it currently stands. Therefore, we invite you to submit a revised version of the manuscript that addresses the points raised during the review process.

**Response:** We would like to sincerely thank you and the editorial team at PLOS Digital Health for the time and effort invested in handling our manuscript. We are grateful for your careful reading, for identifying suitable reviewers, and for coordinating the peer review process.

We are delighted to learn that the reviewers found our manuscript to have merit and that it will be accepted once we address their constructive comments. We greatly appreciate the opportunity to revise and improve our work in line with their feedback.

**Reviewers' Comments:**

**Reviewer's Responses to Questions**

**Comments to the Author**

1. **Comment:** Does this manuscript meet PLOS Digital Health’s publication criteria? Is the manuscript technically sound, and do the data support the conclusions? The manuscript must describe methodologically and ethically rigorous research with conclusions that are appropriately drawn based on the data presented.

**Reviewer #1:** Yes

**Reviewer #2:** Yes

**Reviewer #3:** Partly

**Reviewer #4:** Yes

**Response:** We sincerely thank all four reviewers for their thoughtful evaluation of our manuscript. We are happy to learn that the reviewers have judged the study to meet PLOS Digital Health’s publication criteria. We greatly appreciate this collective recognition of the methodological and ethical rigor of our work, and we value the constructive feedback provided. In this revision, the manuscript has been further refined and improved in line with all the suggestions provided.

2. **Comment:** Has the statistical analysis been performed appropriately and rigorously?

**Reviewer #1:** Yes

**Reviewer #2:** I don't know

**Reviewer #3:** Yes

**Reviewer #4:** Yes

**Response:** We sincerely thank all four reviewers for their assessment of the statistical analysis. We are happy to learn that the reviewers have confirmed the analysis was performed appropriately and rigorously. The revised manuscript provides additional clarity and transparency in the statistical procedures to reinforce robustness.

3. **Comment:** Have the authors made all data underlying the findings in their manuscript fully available (please refer to the Data Availability Statement at the start of the manuscript PDF file)? The PLOS Data policy requires authors to make all data underlying the findings described in their manuscript fully available without restriction, with rare exception. The data should be provided as part of the manuscript or its supporting information, or deposited to a public repository. For example, in addition to summary statistics, the data points behind means, medians and variance measures should be available. If there are restrictions on publicly sharing data—e.g. participant privacy or use of data from a third party—those must be specified.

**Reviewer #1:** No

**Reviewer #2:** No

**Reviewer #3:** Yes

**Reviewer #4:** Yes

**Response:** Many thanks. We confirm that all data underlying the findings in our manuscript have been made fully available in accordance with the PLOS Data Policy. Specifically, we have uploaded the raw data as supplementary materials alongside this revised manuscript. These files include the individual data points underlying the summary statistics (means, medians, and variance measures) reported in the study.

4. **Comment:** Is the manuscript presented in an intelligible fashion and written in standard English?

PLOS Digital Health does not copyedit accepted manuscripts, so the language in submitted articles must be clear, correct, and unambiguous. Any typographical or grammatical errors should be corrected at revision, so please note any specific errors here.

**Reviewer #1:** Yes

**Reviewer #2:** Yes

**Reviewer #3:** Yes

**Reviewer #4:** Yes

**Response:** Many thanks. We appreciate the reviewers’ assessment of the manuscript’s clarity and language. All four reviewers have confirmed that the manuscript is presented in an intelligible fashion and written in standard English. In preparing this revised version, we have carefully reviewed the text to correct minor typographical and grammatical errors, ensuring that the language remains clear, correct, and unambiguous in accordance with PLOS Digital Health requirements.

**Review Comments to the Author**

**Reviewer #1: Overall Assessment**

1. **Comment:** This is a strong and well-structured manuscript that provides genuine theoretical and empirical value. The integration of the Technology Acceptance Model (TAM) with a carefully selected set of Technology–Organization–Environment (TOE) constructs is well justified and substantially improves explanatory power compared to TAM alone. The PLS-SEM analysis is executed rigorously, the psychometric properties are excellent, and the R² values especially 74% for Behavioral Intention are considerably higher than typical TAM-only healthcare studies. The contextual focus on Palestinian governmental and nongovernmental hospitals adds important relevance for digital health adoption in resource constrained environments.

**Response:** We sincerely thank reviewer 1 for the time and efforts spent reading, reviewing, and commenting on our submitted manuscript. Your thoughtful and encouraging assessment are highly appreciated. We greatly appreciate your recognition of the theoretical and empirical contributions, particularly the integration of the Technology Acceptance Model (TAM) with Technology-Organization-Environment (TOE) constructs. Your positive remarks on the rigor of our PLS-SEM analysis, the strength of the psychometric properties, and the high explanatory power of our model are very motivating. We are also grateful for your acknowledgment of the contextual relevance of our study in Palestinian governmental and nongovernmental hospitals, which we believe adds meaningful value to the digital health adoption literature in resource-constrained environments.

2. **Comment:** However, several unexpected or contradictory structural paths are under-discussed or omitted entirely in the Discussion, which currently limits the paper’s interpretive depth and theoretical contribution. These findings are not routine non-supports; they represent potentially important mechanisms in resource-limited digital health settings and deserve explicit examination. Addressing these points will significantly enhance the manuscript’s impact. Therefore, I recommend Major Revision.

**Response:** Many thanks for your valuable feedback. In this revision, we have carefully addressed all the comments and suggestions regarding the unexpected or contradictory structural paths. As you have suggested, we have expanded the Discussion section to explicitly examine these findings, highlighting their potential mechanisms and implications for digital health adoption in resource-limited settings in this revision. We believe this deeper analysis enhances the interpretive depth and theoretical contribution of the study. Please see below how each of your comments was addressed and how the manuscript was revised accordingly.

**Major Compulsory Revisions**

3. **Comment:** Unexpected or Negative Path Coefficients Need Dedicated Discussion. Several structural paths are surprising, counterintuitive, or theoretically meaningful but receive little or no discussion:

- Compatibility → PU: β = –0.14, p = .05 (borderline significant negative)
- TMS → PEOU: non-significant
- ITS → PEOU: strongly non-significant

These results challenge conventional TAM/TOE relationships and may reflect important contextual dynamics in Palestinian health systems. For example: The negative Compatibility → PU path may indicate that systems closely matching existing (often paper-based or inefficient) workflows are perceived as less useful, as they automate inefficiency rather than transform it. Some LMIC studies show similar patterns when “too compatible” systems fail to deliver real improvements.

The fact that TMS and ITS significantly influence PU but not PEOU suggests current training/support helps users understand the system’s value but does not reduce perceived effort common when training is didactic rather than hands-on, or when IT support is reactive.

**Action required:** Add a subsection (approx. 300–400 words) in the Discussion explicitly interpreting these findings. These insights could be among the most valuable contributions of the paper.

**Response:** We thank the reviewer for this insightful observation. As you have suggested, we have added a dedicated subsection in the Discussion explicitly interpreting the unexpected and non-significant path coefficients. This new section examines the negative COMP → PU relationship, as well as the non-significant effects of TMS → PEOU and ITS → PEOU, situating them within the contextual realities of Palestinian hospitals and comparable LMIC studies. We argue that these findings highlight how compatibility with inefficient legacy workflows may reduce perceived usefulness, and how current managerial and IT support practices emphasize strategic value rather than ease of use. We believe this expanded discussion strengthens the manuscript by offering theoretically meaningful insights into EHIS adoption in resource-limited settings. Please see **Lines 506-353.**

4. **Comment:** Missing Path: Complexity → PEOU

The model includes Complexity → PU but omits the standard Complexity → PEOU path widely reported in TAM literature.

**Action required:** Either (1) justify the omission explicitly, or (2) test and report the CPLX → PEOU path post-hoc (even if non-significant). This will reinforce confidence in the model specification.

**Response:** We thank the reviewer for highlighting this important point. As you have suggested, we have explicitly addressed the CPLX → PEOU path in the manuscript. A methodological justification for its omission has been added under the Methods: Model refinement process subsection. Please see **Lines 295-304.**

In addition, a dedicated note has been included in the Discussion to interpret its theoretical relevance and contextual implications. Please see **Lines 536-543.**

5. **Comment:** Data Availability Statement Not Compliant With PLOS Requirements. The current statement (“data available upon reasonable request”) does not comply with PLOS Digital Health’s mandatory open data policy.

**Action required:** Deposit the anonymized dataset and/or the indicator correlations, means/SDs, and PLS-SEM bootstrapping output in a public repository (e.g., Zenodo, Figshare, Dryad) and update the Data Availability Statement accordingly.

**Response:** Many thanks for this observation. In this revision, we have added the raw data as Supplementary information. We confirm that all data underlying the findings in our manuscript have been made fully available in accordance with the PLOS Data Policy. Specifically, we have uploaded the raw data as supplementary materials alongside this revised manuscript. These files include the individual data points underlying the summary statistics (means, medians, and variance measures) reported in the study.

6. **Comment:** Missing Structural Model Diagram (Figure 1). Figure 1 is referenced several times but absent from the PDF.

**Action required:** Include the full structural model diagram with standardized coefficients, significance markers, R² values, and f² where applicable.

**Response:** Many thanks for this comment. As you suggested, Figure 1 shows that depicts a conceptual paradigm integrating the TAM and the TOE framework. Please see **Figure 1** and **Lines 888-897.**

In addition, Figure 2 in the revised manuscript presents the full structural model with standardized coefficients, significance markers, R^2^ values, and f^2^ where applicable. Please see **Figure 2** and **Lines 898-901.**

7. **Comment:** Add a Summary Table of Hypothesis Testing. Results are currently dispersed across text.

**Action required:** Add a table summarizing all hypotheses, including:

hypothesis number, path, β, t-value, p-value, f², and supported/not supported.

This is standard in PLS-SEM publications and improves readability.

**Response:** We thank the reviewer for this suggestion. As you suggested, we have presented all hypotheses (H1-H15) along with their paths, standardized coefficients (β), p-values, and supported/not supported status in Table 6. Please see **Table 6** and **Lines 867-870.**

8. **Comment:** Clarify Sampling Details and Response Rate.

The Methods state that 220 healthcare professionals participated, but total questionnaires distributed, recruitment method, and response rate are not reported.

**Action required:**

Add 1–2 sentences clarifying:

- number distributed
- response rate
- sampling method (convenience, purposive, stratified, etc.)
- any potential non-response bias

This enhances transparency.

**Response:** We thank the reviewer for this suggestion. As you suggested, we have provided the number of potential participants initially invited, response rate, and sampling method. Please see **Lines 229-231, 348-349.**

In addition, the participant recruitment process was described in Supplementary Figure. Please see **S1 Fig.**

Again, we have also discussed the potential non-response bias. Please see **Lines 599-601.**

9. **Comment:** Strengthen Sample Size Justification

The manuscript currently foregrounds Raosoft (designed for simple random sampling), which is not ideal for PLS-SEM.

**Action required:** Lead with the PLS-SEM-relevant justifications (inverse square root method, 10-times rule, or PLSpredict).

Raosoft may be retained as secondary or footnoted.

**Response:** We thank the reviewer for this suggestion. As you have suggested, we have re-ordered the sample size calculation method. In this revision, the 10-Times Rule and the inverse square root method are the primary approaches and raosoft is secondary. Please see **Lines 212-231**.

**Minor Compulsory Revisions**

10. **Comment:** Use “ranged from X to Y” rather than “ranged X–Y” when reporting indicator loadings.

**Response:** We thank the reviewer for this suggestion. As you have suggested, ranges were revised from X–Y to X to Y. Please see **throughout the revised manuscript.**

11. **Comment:** Refine minor phrasing for clarity (e.g., “are more likely to perceive it as useful”).

**Response:** We thank the reviewer for pointing this out. As you have suggested, this was revised. Please see **Lines 148-149**.

12. **Comment:** Reduce repetition of “resource-limited” by occasionally using alternatives (“low-resource,” “constrained,” etc.).

**Response:** We thank the reviewer for pointing this out. As you have suggested, in many places, resource-limited was revised to resource-constrained or low-resource. Please see **throughout the revised manuscript.**

13. **Comment:** Double-check that all β values in the abstract match those reported in the Results section.

**Response:** We thank the reviewer for this comment. As you have suggested, values were double checked.

**Optional but Valuable Suggestions**

14. **Comment:** Link the non-significance of Complexity → PU to mandatory-use environments where effort expectancy is less influential.

**Response:** We thank the reviewer for this suggestion. As you have suggested, this was linked. Please see **Lines 543-547**.

15. **Comment:** Add a brief comparison of your R² values with recent LMIC TAM/TOE studies to underscore the strength of your model.

**Response:** We thank the reviewer for this suggestion. As you have suggested, a brief comparison was added. Please see **Lines 535-553**.

16. **Comment:** Highlight the unusually strong competitive pressure effects (some of the highest reported in healthcare TAM literature).

**Response:** We thank the reviewer for this suggestion. As you have suggested, this was highlighted. Please see **Lines 548-553**.

**Summary**

**Comment:** This is a promising and well-constructed manuscript with strong methodological rigor, an excellent theoretical foundation, and important contextual relevance. The unexpected findings particularly the negative Compatibility → PU path and the selective influence of TMS/ITS are potentially the most policy-relevant contributions and deserve fuller exploration. Addressing the points above will significantly strengthen the paper’s theoretical clarity and practical value. I look forward to reviewing a revised version.

**Response:** We sincerely thank the reviewer for their thoughtful and constructive feedback. We are pleased that the reviewer found that the manuscript’s methodological rigor, theoretical foundation, and contextual relevance were recognizable. We have carefully addressed all comments, including fuller exploration of the unexpected findings (Compatibility → PU and the selective influence of TMS/ITS), and strengthened the clarity and practical implications of our work. We are delighted that the reviewer views the revised manuscript favorably and look forward to its acceptance.

**Reviewer #2:**

We sincerely thank reviewer 2 for the time and efforts spent reading, reviewing, and commenting on our submitted manuscript. Your thoughtful and encouraging assessment are highly appreciated. We are happy to learn that the reviewer has found the manuscript timely, important, relevant, and interesting. We are also very happy to learn that the reviewer will accept the manuscript after addressing the comments of the reviewer and revise the manuscript accordingly. Please see below how the manuscript was revised accordingly.

1. **Comment:** Pro 1: Strong methodological validation.

The reviewer notes that one of the major strengths of this study is the thorough validation of the TAM-TOE instrument. The authors apply multiple validation methods—including indicator loadings, Cronbach’s α, composite reliability, AVE, Fornell–Larcker, HTMT, VIF checks, SRMR, and hierarchical regression—to support the reliability and validity of the constructs. The refinement steps are described clearly, and the hierarchical regression analysis demonstrates additional explanatory value beyond demographic variables. The reviewer encourages the authors to keep this level of methodological detail, as it supports the credibility of the findings.

**Response:** We sincerely thank reviewer 2 for recognizing the methodological rigor of our study. We are pleased that the thorough validation of the TAM–TOE instrument—including multiple reliability and validity checks as well as hierarchical regression—was appreciated. We agree that retaining this level of methodological detail enhances the credibility of our findings, and we have ensured that these validation steps remain clearly presented in the revised manuscript.

2. **Comment:** Pro 2: Clear contextual grounding and focused use of extended TOE factors.

The reviewer finds that the authors present an original and context-specific application of the TAM-TOE framework by situating the analysis within Palestinian hospitals facing resource limitations. The authors successfully relate technological and organizational factors—such as competitive pressure, system quality, and compatibility—to the realities of EHIS use in these settings. The reviewer appreciates how the discussion links the statistical results back to practical issues in this context, which strengthens the relevance of the study for local decision-makers and implementers.

**Response:** We sincerely thank the reviewer for recognizing the clear contextual grounding and focused use of extended TOE factors in our study. We are pleased that the application of the TAM–TOE framework to Palestinian hospitals was found to be original and relevant. In line with the reviewer’s encouragement, we have further strengthened the discussion section to more explicitly connect the statistical findings—particularly those related to competitive pressure, system quality, and compatibility—to practical challenges faced in resource-limited healthcare settings. These refinements underscore the policy relevance of our results and enhance the manuscript’s value for local decision-makers and implementers.

3. **Comment:** Con 1: Questionnaire items should be introduced earlier and linked directly to item codes used in the Result.

The reviewer suggests that the authors introduce questionnaire items earlier in the paper. In the current version, item codes such as RA1, CPLX2, and COMP3 appear in the Results section before readers know what the items actually measure. Because the wording of these items only appears later in the appendix or in subsequent tables, it becomes difficult for readers to interpret loadings and reliability values. The reviewer recommends adding a summary table of constructs and items in the Methods section or briefly listing item descriptions when they first appear in the Results.

**Response:** We thank the reviewer for this suggestion. As you have suggested, we have added a new summary table of constructs and items in the Methods section. Please see **Lines 239-240, 845-846 and Table 1.**

4. **Comment:** Con 2: Results section would benefit from clearer organization regarding model refinement

The reviewer suggests reorganizing the Results section for better clarity. Table 2 appears early and seems to contain final results, but the narrative later introduces a refinement stage where several items are removed. This can cause confusion about which results correspond to the initial versus the refined model.

**The reviewer recommends:**

- explaining the refinement process before presenting the final measurement tables, or
- explicitly labeling tables as “initial model” and “refined model.”

This restructuring would help readers follow the sequence of measurement evaluation and refinement more easily.

**Response:** We thank the reviewer for this suggestion. As you have suggested, we have revised the titles of the tables so that one indicates the initial model and the second as the refined model. The removal of some items was justified early in the manuscript. Please see **Lines 849-850, 867.**

**Reviewer #3:**

1. **Comment:** This manuscript presents an important and timely investigation of factors influencing electronic health information system (EHIS) acceptance in resource-limited hospital settings using an extended TAM–TOE framework. The topic is highly relevant to health informatics and health system strengthening, particularly in low- and middle-income countries undergoing digital transformation. The statistical analysis is generally rigorous; the use of PLS-SEM is appropriate for the complexity of the model and the sample size, and the authors provide a comprehensive evaluation of measurement reliability and validity.

**Response:** We sincerely thank Reviewer 3 for the time and effort devoted to reading, reviewing, and commenting on our manuscript. Your thoughtful and encouraging assessment is highly appreciated. We are pleased to learn that you found the study timely, important, and relevant, with rigorous statistical analysis and appropriate use of PLS-SEM. We are also gratified that the reviewer recognizes the comprehensive validation of measurement reliability and validity. We have carefully addressed all comments and revised the manuscript accordingly, and we are happy to know that the reviewer views the manuscript favorably.

2. **Comment:** At the same time, several methodological and contextual clarifications would enhance the transparency and interpretability of the study.

**Response:** We thank the reviewer for the comments and suggestions that enhanced methodological and contextual clarifications, transparency and interpretability. We have carefully addressed all of these points in the revised manuscript. Please see below for a detailed account of the revisions made in response to each comment.

3. **Comment:** Contextual detail regarding hospital types and EHIS systems.

The manuscript notes that participants were recruited from both governmental and nongovernmental hospitals, but additional information regarding the number, size, digital maturity, and geographical distribution of these facilities would help readers better understand the setting. It would also be helpful to clarify whether the participating hospitals used a unified EHIS platform or whether system characteristics varied across sites. Such information would strengthen the contextual grounding of the findings.

**Response:** We thank the reviewer for the comments and suggestions. As you have suggested, we have included a description of the EHIS implemented in Palestinian hospitals. Please see **Lines 197-206.**

4. **Comment:** Departmental or unit-level distribution of participants.

Although the study distinguishes between clinical and nonclinical roles, it does not report respondent distribution across specific departments (e.g., emergency, outpatient clinics, inpatient wards, laboratory, radiology, pharmacy). Given that workflow integration and system usage patterns differ substantially across units, providing this information—or acknowledging its absence—would help contextualize variations in acceptance.

**Response:** We thank the reviewer for raising the issue of departmental or unit-level distribution of participants. Indeed, respondents in our study represented a variety of hospital departments and units, including emergency, outpatient clinics, inpatient wards, laboratory, radiology, and pharmacy. However, these departmental distributions were not included in the statistical comparisons, as our analysis focused on clinical versus nonclinical roles rather than unit-level differences. We have now acknowledged this in the Limitations section, noting that future studies should examine departmental variations more explicitly, since workflow integration and system usage patterns may differ substantially across units. Please see **Lines 601-607.**

5. **Comment:** Connection between empirical findings and practical implications.

While the study identifies several strong predictors of EHIS acceptance (e.g., system quality, competitive pressure, PEOU, PU), some recommendations in the Implications section appear somewhat general and not fully aligned with the empirical results. For instance, competitive pressure showed a relatively strong effect, yet the discussion does not elaborate on how such dynamics manifest in the Palestinian hospital context. Strengthening this linkage would improve the relevance and specificity of the practical implications.

**Response:** We thank the reviewer for this comment. As you have suggested, this was added to the implications. Please see **Lines 584-589.**

**Recommendation: Minor Revision**

6. **Comment:** Overall, this is a well-designed study with strong methodological foundations and meaningful contributions to the literature. The issues noted above primarily require clarification rather than substantial reanalysis. Therefore, I recommend Minor Revision, with attention to additional contextual detail and strengthened interpretation to maximize the clarity and utility of the manuscript.

**Response:** We sincerely thank the reviewer for the positive and encouraging assessment of our manuscript. We are pleased that the study was recognized as well-designed, methodologically rigorous, and contextually meaningful. We have carefully addressed all the clarifications requested, including the addition of contextual detail and strengthened interpretation, to maximize transparency and utility. We are happy to know that the reviewer recommends Minor Revision and will accept the manuscript once these points are addressed.

**Reviewer #4:**

1. **Comment:** Manuscript Number: PDIG-D-25-00865

Title: Adopting and validating a technology acceptance model-based paradigm to assess acceptance and satisfaction with electronic health information system by healthcare providers in resource-limited governmental and nongovernmental hospitals

Recommendation: Major Revision

**Response:** We sincerely thank Reviewer 4 for the time and effort devoted to reading, reviewing, and commenting on our manuscript. Your thoughtful feedback and constructive recommendations are highly appreciated. We acknowledge the recommendations provided by the reviewer and have carefully addressed the points raised to strengthen the clarity, contextual detail, and interpretive value of the study. We are grateful for your engagement with our work and look forward to your assessment of the revised manuscript.

**Reviewer Summary**

2. **Comment:** This manuscript presents a study validating an extended Technology Acceptance Model (TAM) integrated with the Technology-Organization-Environment (TOE) framework to assess the adoption of Electronic Health Information Systems (EHIS) in Palestinian hospitals. The study addresses a significant gap in the literature regarding digital health implementation in resource-constrained environments. The authors are to be commended for conducting rigorous data collection in a challenging setting. The choice of Partial Least Squares Structural Equation Modeling (PLS-SEM) is methodologically appropriate for the exploratory nature of the model, and the statistical reporting of validity metrics is generally robust.

**Response:** We greatly appreciate your recognition of the study’s contribution in validating an extended TAM–TOE framework for EHIS adoption in Palestinian hospitals, and your commendation of the rigorous data collection in a challenging setting. We are pleased that the methodological choice of PLS‑SEM and the robustness of our validity reporting were found appropriate and well‑executed. We have carefully addressed the clarifications and recommendations noted in your comments, and we look forward to your assessment of the revised manuscript.

3. **Comment:** However, there are substantial concerns regarding the interpretation of null results, the transparency of the sampling procedure, and the structural organization of the manuscript. Specifically, the conclusion that a non-significant finding implies "user resilience" is methodologically unsound and requires revision. I recommend Major Revisions to address the following points.

**Response:** We acknowledge the recommendations provided by the reviewer and have carefully addressed the points raised to strengthen the clarity, contextual detail, and interpretive value of the study. We are grateful for your engagement with our work and look forward to your assessment of the revised manuscript.

**Major Comments**

4. **Comment:** Clarification of Sampling Procedure and Response Rate

The manuscript states that the required sample size was calculated to be exactly 220 participants using the Raosoft calculator. The Results section subsequently reports that exactly 220 healthcare providers participated in the study. It is statistically unusual for the final number of valid, analyzed responses to match the calculated minimum threshold exactly, unless a specific stopping rule was applied or the response rate was 100%. To ensure reproducibility and transparency regarding potential selection bias, please clarify the data collection process. Specifically, did data collection cease immediately once the target of N=220 was reached, and were there any incomplete or excluded surveys? Please also report the total number of healthcare providers approached versus those who participated (i.e., the response rate).

**Response:** We thank the reviewer for raising this important point regarding sampling procedure and response rate. In the revised manuscript, we have clarified the data collection process in detail. Specifically, we have reported the total number of healthcare providers invited to participate, the number of completed surveys, and the final response rate. We also explained that data collection did not cease immediately upon reaching the calculated minimum sample size, and that incomplete or invalid surveys were excluded from the analysis. To further enhance transparency, we have added a new flow diagram illustrating the recruitment process, exclusions, and final sample size. Please see **Lines 229-231, 348-349 and S1 Fig.**

5. **Comment:** Interpretation of Null Results regarding Complexity (CPLX).

In the Conclusion, the manuscript states that the non-significant effect of Complexity on Usefulness (P=.46) "highlight[s] the resilience of user attitudes". Drawing a specific psychological conclusion, such as user resilience, from a null statistical result is methodologically unsound. A failure to reject the null hypothesis indicates an absence of evidence for the relationship in this specific sample; it does not empirically validate the presence of "resilience," a construct that was not measured in the model. Please remove this speculative claim. Instead, discuss this null result in the context of the sample demographics. As shown in Table 1, participants reported a mean of 10.4 years of computer/EHIS usage. It is highly probable that this extensive experience—rather than "resilience"—mitigated the perceived effect of system complexity.

**Response:** Many thanks for this comment. As you have suggested, the Discussion and Conclusion were revised to address this. Please see **Lines 495-500.**

6. **Comment:** Substantiation of the "Gap" in Related Work.

In the "Related Work" section, the manuscript states that hybrid TAM-TOE frameworks "tend to underperform" when deployed in resource-limited environments. This assertion is the central justification for developing the extended model used in this study. However, the text does not currently provide specific comparative evidence to support this claim, such as citing lower R^2 values from specific previous studies in low-income versus high-income settings. Please strengthen the rationale for the study by citing specific examples or metrics that demonstrate this "underperformance." Providing concrete evidence that previous models fail to capture sufficient variance in developing contexts would make the argument for your extended model much more compelling.

**Response:** Many thanks for this comment. As you have suggested, the Discussion and Conclusion were revised to address this. Please see **Lines 111-117.**

7. **Comment:** Structural Coherence of the Introduction and Background The current organization of the opening sections disrupts the narrative flow of the manuscript. The text presents a "Background" section followed by a separate "Introduction" header, which creates redundancy. Furthermore, the narrative in these sections occasionally relies on emotive or "journalistic" phrasing (e.g., "disappointingly low", "falls short") rather than precise, objective descriptions. Please merge the "Background" and "Introduction" sections into a single, cohesive introductory section. Additionally, please refine the language to be more objective, providing specific adoption percentages or citation-backed data rather than qualitative descriptors.

**Response:** We thank the reviewer for pointing out the structural and stylistic issues in the opening sections. In the revised manuscript, we have merged the “Background” and “Introduction” into a single, cohesive introductory section to improve narrative flow and avoid redundancy. We also refined the language to ensure objectivity, replacing qualitative descriptors (e.g., “disappointingly low,” “falls short”). These changes strengthen the clarity and coherence of the introduction and provide a more rigorous foundation for the study rationale. Please see **Lines 59-117.**

**Minor Comments**

8. **Comment:** Causal Language in a Cross-Sectional Design.

The authors correctly acknowledge in the Limitations section that the cross-sectional design precludes causal inferences. However, the language used throughout the Abstract and Results sections is frequently deterministic, using terms such as "SQ enhanced PU," "CP drove PU," and "impact of...". Given that PLS-SEM on cross-sectional data establishes predictive association rather than causality, please revise these verbs to reflect association (e.g., "was associated with," "predicted," "was related to") to ensure scientific accuracy.

**Response:** We thank the reviewer for this valuable observation. In the revised manuscript, we carefully reviewed the Abstract, Results, and related sections to ensure that the language accurately reflects the cross‑sectional design and the predictive nature of PLS‑SEM. Deterministic verbs such as “enhanced,” “drove,” and “impact” have been replaced with more appropriate terms, including “was associated with,” “predicted,” and “was related to.” Please see **throughout the revised manuscript.**

9. **Comment:** Model Parsimony and Theoretical Overlap.

The proposed research model is extensive, incorporating seven distinct exogenous predictors and 15 hypothesized paths. While the statistical discriminant validity appears adequate, there is significant theoretical overlap between constructs such as Relative Advantage (RA) versus System Quality (SQ), or Compatibility (COMP) versus Perceived Ease of Use (PEOU). Please expand the Discussion section to address the limitation of using such a high-dimensional model. Specifically, discuss whether the inclusion of so many predictors might dilute the explanatory power of individual factors or obscure the most critical drivers of adoption. Acknowledging the trade-off between model complexity and parsimony would strengthen the manuscript.

**Response:** We thank the reviewer for this insightful observation. In the revised manuscript, we have added a new paragraph in the Limitations section explicitly acknowledging the trade‑off between model complexity and parsimony. We note that while our extended model incorporated seven exogenous predictors and 15 hypothesized paths, potential theoretical overlap exists between constructs such as Relative Advantage versus System Quality, and Compatibility versus Perceived Ease of Use. Although discriminant validity was established statistically, we recognize that including multiple predictors may dilute the explanatory power of individual factors or obscure the most critical drivers of EHIS adoption. We have therefore highlighted this limitation and suggested that future research explore more parsimonious models to balance comprehensiveness with theoretical clarity. Please see **Lines 609-615.**

Once again, we would like to thank you and the reviewers for the constructive feedback which we think have significantly improved the quality, clarity, and flow of the revised manuscript.

Having addressed all reviewers’ comments and revised the manuscript accordingly, we are looking forward to hearing from you soon.

Best regards

Ramzi Shawahna
